# Assessment of Organic Pollutants Desorbed from Plastic Litter Items Stranded on Cadiz Beaches (SW Spain)

**DOI:** 10.3390/toxics13080673

**Published:** 2025-08-09

**Authors:** Juan Manuel Traverso-Soto, Manuel Figueredo, Irene Punta-Sánchez, Olivia Campana, Elisabetta Ciufegni, Miriam Hampel, Joana Buoninsegni, Manuel A. Manzano Quiñones, Giorgio Anfuso

**Affiliations:** 1 Instituto Universitario de Investigación Marina (INMAR), Campus de Puerto Real, Universidad de Cádiz, 11519 Cádiz, Spain; juanmanuel.traverso@uca.es (J.M.T.-S.); manuel.manzano@uca.es (M.A.M.Q.); 2 Instituto de Investigación Vitivinícola y Agroalimentaria (IVAGRO), Campus de Puerto Real, Universidad de Cádiz, 11519 Cádiz, Spain; 3 Department of Environmental and Prevention Sciences, University of Ferrara, 44122 Ferrara, Italyjoana.buoninsegni@unife.it (J.B.); 4 Department of Ecology and Coastal Management, Institute of Marine Sciences of Andalusia (ICMAN—CSIC), Campus Universitario Río San Pedro, 11519 Puerto Real, Spain; miriam.hampel@uca.es

**Keywords:** coastal monitoring, leaching, organic pollutants, FTIR, GC–MS

## Abstract

This paper constitutes a preliminary study that evaluates the organic pollutants desorbed from “fresh” plastic litter, i.e., recently stranded items, on three beaches in Cadiz (SW Spain): Bajo de Guia, La Jara, and La Puntilla. Beach litter items were collected and classified in laboratory according to their composition and use. Leachates were obtained by stir bar sorptive extraction (SBSE) and analysed with gas chromatography–mass spectrometry (GC–MS). Fifty-five target organic compounds—including polycyclic aromatic hydrocarbons (PAHs), pesticides, fragrances, insect repellents, and UV filters—were quantified. Plastics accounted for the majority of litter by both number and weight. Cigarette butts and wipes were also prevalent and served as key sources of leachable PAHs. With respect to the main pollutants found in plastic films, hard plastics, and wipes, fragrances such as OTNE1 (1-Tetramethyl Acetyloctahydronaphthalene), OTNE2 (2-Tetramethyl Acetyloctahydronaphthalene), DEET (N, N-Diethyl-Meta-Toluamide), galaxolide, and tonalide were dominant, with concentrations exceeding 100 ng/g in some cases. DEET was the most common insect repellent detected. These findings underscore the role of beach litter, especially plastic waste, as a vector for persistent and emerging organic pollutants, highlighting the urgent need for improved waste management and monitoring practices to mitigate ecological risks associated with plastic pollution.

## 1. Introduction

Marine litter is “any persistent, manufactured or processed solid material discarded, disposed or abandoned in marine and coastal environments” [1]. Beach litter arrives from land (ca. 80%) through rivers, wind, and run off and/or is directly discharged by beach users, and from the sea (ca. 20%) because of shipping and fishing activities, accidental spills during transportation, etc. [1,2,3]. Despite its origin, it is mostly (50–80%) constituted by plastic materials [4,5,6]. Marine environments are the ultimate sink for a wide range of plastic polymers, and it is estimated that over 10 million tons of plastics yearly end up in the oceans via various pathways [7,8]. Common types of plastics include polyethylene (PE), found in items like plastic bottles, bags, and pipes; polypropylene (PP), used in straws, bottle caps, and milk containers; polyvinyl chloride (PVC), which appears in drainage pipes, window frames, and water supply lines; polystyrene (PS), present in food packaging, foam cushioning, and toys; and polyethylene terephthalate (PET), often used in microwavable containers, carbonated beverage bottles, furniture, and pillows [9]. The great abundance of plastic items in marine environments is linked to its large use and durability [10] and, additionally, to the capacity of being transported long distances by marine currents, such as large oceanic gyres [11]. Their accumulation on the shore depends on the morphodynamic characteristics of each beach, as well as its location and exposure to wave action and marine currents.

In natural environments, plastics suffer degradation or aging, primarily driven by physicochemical transformations [12], with biodegradation playing a minor role. The extent of the abiotic degradation depends on factors such as temperature, UV light intensity, and/or the mechanical forces acting on the material. In particular, the plastics stranded on beaches often face higher temperatures than those occurring in open waters, converting it to a critical factor of degradation in these scenarios [13]. The aging process favours the release of plastic fragments and components into the environment, such as water-soluble organic compounds or volatile molecules (e.g., short-chain hydrocarbons) [14]. Plastic additives, such as persistent organic pollutants (POPs) (e.g., plasticisers, flame retardants, antioxidants, acid scavengers, lubricants, pigments, antistatic agents, slip agents, or thermal light stabilisers), confer specific properties to enhance plastic functionality. However, they are generally not chemically bound to the polymers and can leach out as the plastic ages and degrades [15,16], becoming bioavailable to organisms [17,18]. Beyond additives, POPs can also be adsorbed onto plastics in the environment [19,20]. Therefore, plastic items may accumulate hydrophobic persistent organic pollutants to concentrations several orders of magnitude higher than in the surrounding water [21]. These compounds can be re-released into the environment during degradation or can interact with organisms through accidental ingestion or a process called leaching, during which contact between a solid (in this case, plastic waste) and a liquid solvent (seawater) leads to the separation of one or more solutes (organic contaminants).

Leaching experiments are characterised by factors such as the liquid–solid (L/S) ratio, the physical properties of the material, the leaching liquid, and the operating conditions, all of which influence the kinetics and equilibrium, thereby affecting the mobility of species from the solid phase to the liquid phase. To simulate plastic leaching in seawater, different experimental setups have been developed to approximate real environmental conditions [14,22,23]. However, although the migration of contaminants from commercial plastics to seawater has been extensively studied, current knowledge about leachates released from aged plastic debris in seawater is very limited [24], particularly when leachate assays involve both agitation and solar exposure experimental conditions.

The assessment of organic pollutants adsorbed onto plastic debris surfaces is an issue of growing environmental concern. Numerous studies have focused on understanding the transformations of plastic materials during environmental exposure [22] or on the determination of dissolved organic carbon (DOC) released during plastic degradation. However, to the best of our knowledge, few studies have specifically addressed the identification of organic compounds that can desorb from plastic debris into the surrounding environment [24]. A powerful analytical approach for the identification and quantification of these compounds is the use of stir bar sorptive extraction (SBSE), which enables the extraction of moderately to highly hydrophobic contaminants from water. In this technique, a magnetic stir bar coated with polydimethylsiloxane (PDMS) adsorbs organic pollutants, providing high preconcentration efficiency and achieving lower detection limits compared to conventional methods [25,26]. The concentrated analytes can then be identified and quantified by gas chromatography coupled to mass spectrometry (GC–MS) [26] that enables the detection of organic pollutants with very high sensitivity levels and is also combined with cutting-edge techniques such as machine learning to establish correlations within large volumes of data [27].

Once incorporated into organisms, organic contaminants can accumulate in tissues, interact with macromolecules, and induce specific effects such as behavioural changes, metabolic disruptions, oxidative stress, and endocrine disruption [28]. As awareness grows, so do concerns about the role that plastic debris plays, not only as a pollutant itself, but also as a carrier for harmful organic substances, highlighting the need for a comprehensive understanding of how these pollutants move through various environments and systems [29].

Previous studies on beach litter along the coast of Cádiz (SW Spain) were limited to the characterisation and quantification of stranded litter items. The present paper aims to assess organic pollutants desorbed from “fresh” plastic litter items (i.e., plastic litter recently transported to the shore by marine/coastal processes) [30] stranded on different beaches along the investigated coastline. Therefore, the present paper deals with a topic that has never been investigated before along the study area: Plastic items from three beaches off the coast of Cadiz were collected and characterised by FTIR analysis; then, a leaching extraction method using SBSE coupled to GC—MS was applied in order to determinate a wide range of regulated and emerging organic pollutants (55 compounds in total, including several types of pesticides, PCBs, PAHs, fragrances, and UV filters) adsorbed in recollected plastic items.

## 2. Materials and Methods

### 2.1. Study Area

**Figure 1:**
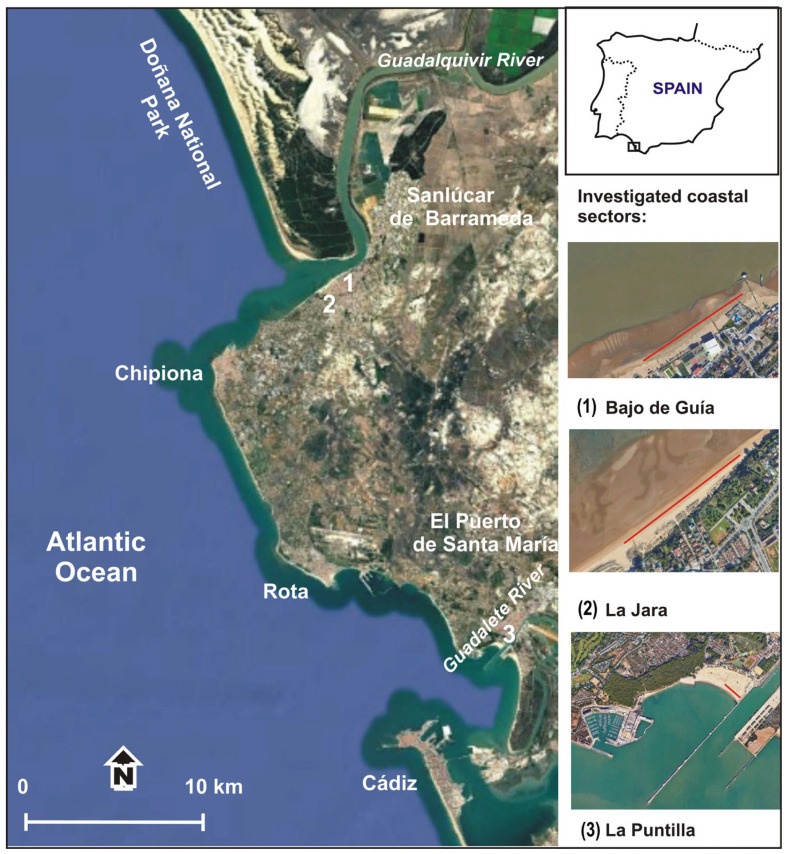
Location map of the beaches investigated with analysed sectors marked in red. Geographic coordinates: Bajo de Guía (latitude 36°47′40″ N, longitude 6°21′50″ W); La Jara (36°46′21″ N, 6°22′45″ W); La Puntilla (36°35′07″ N, 6°14′24″ W).

This study covers three coastal sectors in the Cadiz province on the Atlantic side of Andalucía, southwest Spain (Figure 1), two located in the municipality of Sanlúcar de Barrameda and the other in El Puerto de Santa María. The investigated coast is a mesotidal environment with a tidal range between 2 and 4 m and is influenced by both westerly and easterly winds. Westerly Atlantic low-pressure systems give rise to significant rainfalls and marine storms. East to southeast winds, which blow from the Mediterranean Sea, give rise to small waves because of the limited fetch [31]. All investigated sectors are composed by fine, quartz-rich sand that give rise to smooth, dissipative beach conditions.

The sectors at Bajo de Guía and La Jara beaches, both located in the Sanlúcar de Barrameda municipality, were respectively 400 and 320 m in length. La Jara is located in a wave-exposed coastal area, and Bajo de Guía is in a sheltered beach protected by the sand spit of Doñana and is strongly affected by the Guadalquivir River hydrodynamic processes. The Guadalquivir River, which is the most relevant river on the Atlantic side of Andalucía due to its considerable flow and sedimentary supplies to the coast, stretches for 657 km and flows through large cities such as Sevilla and Cordoba.

The sector at La Puntilla beach, located in El Puerto de Santa María municipality, at the Guadalete River’s mouth, was 170 m in length. La Puntilla is a sheltered area (Figure 1) since it is located at the end of a “funnel-like” marine water body enclosed between the coastline and the northern jetty at the mouth of the Guadalete River [30]. This river presents a length of 172 km and flows through different towns as Jerez de la Frontera and El Puerto de Santa María. According to Williams et al. [32], both the Guadalquivir and the Guadalete rivers provide solid waste materials to the coast. Local authorities carry out daily mechanical and manual clean-up operations at the study sectors from April to September, and manual beach cleaning is usually carried out on a daily/weekly frequency from October to March.

Both Sanlúcar de Barrameda and El Puerto de Santa María are major beach tourist destinations, driven by coastal appeal and favourable weather conditions during many months of the year. In 2022, Sanlúcar contained ca. 70,000 inhabitants and 220,000 tourists, and El Puerto de Santa María had around 90,000 residents and recorded ca. 700,000 visitors [33].

### 2.2. Sampling Methodology

In order to only recollect and analyse “fresh” litter items, which refers to litter recently stranded on the shore by marine/coastal processes [30], the surveys were carried out during morning low-tide conditions on 7 November 2024 at Sanlúcar de Barrameda beaches and on 3 March 2025 at El Puerto de Santa María in a strip of 5 m in width along the strandline [34], i.e., a strip extended 2.5 m both landward and seaward of the strandline. Such line corresponds with the last high-tide mark and usually contains most of the beach litter and vegetation debris stranded during the previous days, as observed in other studies [32,35]. In this paper, which constitutes a preliminary investigation, the separation between “fresh” and “deposited” litter was based on the experience of the surveyors, and quantifiable measures of the age of recollected plastic items were not carried out. Such aspect is relevant because the aging process of plastic items can determine the quantity and composition of leachates. Collected litter was stored with labels indicating the collection date and the name of the beach. In order to avoid potential contamination among different types of items, each litter category (e.g., plastic films, hard plastics, cigarette butts, wet wipes, etc.) was stored separately. Afterward, in the laboratory, litter items were cleaned of sand and algae, dried, weighed, and finally classified following the Joint List of Litter Categories for Macrolitter Monitoring adopted by the Marine Strategy Framework Directive (MSFD) Coordination Group [36]. The classification is based on a hierarchical system to classify objects by material (chemicals, textiles, organic materials, glass and ceramics, metals, plastics, paper and cardboard, rubber, and wood), function (e.g., fishing- and food-related), and size (2.5–50 cm and >50 cm).

The number of items and the total weight of each category were recorded per beach, and data were normalised according to the length of each of the investigated beach sectors. This normalisation enabled the direct comparisons of litter abundance (number m^−1^) and density (grams m^−1^) across different beaches.

### 2.3. Sample Preparation for FTIRAnalysis on La Puntilla Beach Litter

Samples collected from La Puntilla beach were sorted into different groups to facilitate molecular identification. These categories included cigarette butts, dust wipes, wet wipes, sanitary towels, hard plastics, and plastic films. The most representative items from each category were selected and preliminarily cleaned to remove the majority of adhered debris (sediment, vegetable debris, etc.).

**Figure 2:**
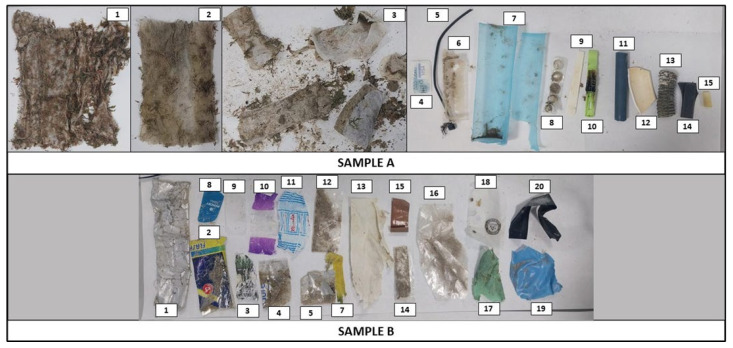
Macro litter items that were identified by FTIR spectroscopy. The labels were used to identify each fragment for FTIR analysis in the results section (see Table 1 and Figures S1 and S2).

Selected macro-litter items (2.5–50 cm) were photographed and assigned a unique identification code prior to the removal of a fragment measuring between 0.5 and 2.5 cm (Figure 2). Each fragment was placed in a plastic Petri dish, adhered to the surface by the addition of a single drop of Milli-Q water.

The collected items were chemically identified using Fourier Transform Infrared (FTIR) spectroscopy. Infrared spectra were recorded employing a PerkinElmer Spectrum 3 FT-IR Spectrometer with a diamond crystal GladiATR Vision accessory (PerkinElmer, UK). The scanners range from 4000 to 650 cm^−1^, with a nominal resolution of 4 cm^−1^. For each analysis, 32 scanners were accumulated for the background and 4 scanners for each sample. Infrared spectra of the sampled items were compared with reference spectra from the supplier’s libraries (PerkinElmer) using SpectrumIR software (Spectrum IR10.7.2). Key vibrational bands serving as diagnostic fingerprints for polymer identification were selected based on guidelines established elsewhere [37]. Furthermore, the identification of other organic components, such as cellulose, relied on their characteristic infrared absorption frequencies [38,39,40]. Spectra exhibiting a singular, broad absorption feature within the 1000–1050 cm^−1^ range, consistent with COC stretching vibrations in polysaccharides, were visually inspected and classified as cellulose fragments.

### 2.4. Materials, Reagents, and Chemicals

Methanol and ethyl acetate, both of chromatography quality, and sodium chloride were purchased from Scharlab (Barcelona, Spain). Commercial polydimethylsiloxane (PDMS) stir bars (size 10 mm × 0.5 mm, length × film thickness) were purchased from Andaluza de Instrumentación, S.L. (Sevilla, Spain).

Mixtures of polycyclic aromatic hydrocarbons (PAHs) (acenaphthylene, acenaphthene, fluorene, anthracene, phenanthrene, pyrene, fluoranthene, benzo[a]anthracene, chrysene, benzo[b]fluoranthene, benzo[k]fluoranthene, and benzo[a]pyrene), polychlorinated biphenyls (PCB28, PCB52, PCB138, PCB153, PCB180, and PCB101), and a deuterated PAH mixture (chrysene d12, phenanthrene d10, and acenaphthene d10) were purchased from Dr. Ehrenstorfer GmbH (Augsburg, Germany). Triclosan (TCS), methyl-triclosan (MTCS), 2-ethylhexyl-4-methoxycinnamate (EHMC), 4-methylbenzylidene camphor (4-MBC), N,N-diethyl-meta-toluamide (DEET), galaxolide, tonalide, OTNE fragrances (1, 2), pyrethroids (bifenthrin, permethrin (I.II)), organochlorine pesticides (lindane, heptachlor, heptachlor epoxide, α-endosulfan, β-endosulfan, endosulfanSulfate, o,p’-DDT, p,p’ DDT, o,p’-DDE, dieldrin, endrin, methoxyclor, and endrin ketone), and organophosphorus pesticides (parathion, ethion, carbophenothion, and chlorpyrifos) were purchased from Sigma–Aldrich (Madrid, Spain). Triazines (ametryn, secbumeton, prometryn, terbutryn, propazine, and terbuthylazine) were purchased from LGC Standards (Barcelona, Spain). Stock solutions of these analytes were prepared in methanol and stored at −20 °C in tightly closed amber vials.

### 2.5. Litter Leaching Through SBSE Extraction

The organic compounds’ extraction is based on a methodology found elsewhere [26]. Briefly, analytes were extracted from “fresh” litter items, collected from the beaches mentioned above, by stir bar sorptive extraction (SBSE). The aim of this extraction process was to maximise the sorption of analytes adsorbed in different litter categories, separately stored. The quantity of items used (10 g) was representative of the different categories of studied materials and, quite often, represented the totality of each of them, i.e., cigarette butts at Bajo de Guia. Therefore, it was not possible to carry out replicates. First, 10 g of selected cigarette butts, plastics with different hardness (film and hard), and wet wipes, were mixed with 200 mL of Milli-Q water in order to obtain leachates from each litter category. Prior to use, all PDMS stir bars were preconditioned by soaking them in a mixture of acetonitrile/methanol (80:20, *v*/*v*) overnight. Later, these bars were placed in amber glass flasks containing the leachates (200 mL) and 100 g L^−1^ of sodium chloride and stirred at 900 rpm during 72 h at room temperature, with the objective of maximising the sorption of target compounds on PDMS bars. Later, the obtained concentrations of target compounds detected in leachates were normalised with respect to the total mass of materials analysed (ng g^−1^).

Surrogates (chrysene d12, phenanthrene d10, and acenaphthene d10) were also added to these samples (0.1 ng mL^−1^) to determine possible fluctuations during the extraction and analysis procedures. After extraction, the PDMS bars were gently dried, and analytes were desorbed by liquid desorption. PDMS bars were sonicated for 30 min in 2 mL vials filled with inserts containing 200 µL of ethyl acetate. Then, 1 µL of each sample was injected into a gas chromatography system.

### 2.6. Triple Quadrupole Mass Spectrometry Detection

The analysis of compounds extracted from leachates was carried out using a gas chromatograph coupled to a triple quadrupole mass spectrometer (GC–MS TQ8040; Shimadzu Scientific Instruments, Kyoto, Japan). Separation was achieved using a BPX5 capillary column (30 m × 0.25 mm i.d., 0.25 μm film thickness, SGE Analytical Science, Ringwood, Australia). Helium was used as the carrier gas under constant linear velocity (35.0 cm s^−1^). The oven temperature program was as follows: initial temperature of 70 °C (held for 1.00 min), increased at 25 °C min^−1^ to 160 °C, followed by a ramp of 3 °C min^−1^ to 220 °C (held for 10.00 min), and finally 2 °C min^−1^ to 250 °C (held for 5.00 min). The injection was performed in splitless mode at 250 °C.

Mass spectrometer was programmed in multiple reaction monitoring (MRM) mode. The ion source temperature was set at 200 °C and the interface temperature at 250 °C. Compounds were monitored using compound-specific transitions across their respective retention time windows.

Quantification of the target compounds was performed using external calibration curves. Calibration standards were prepared in Milli-Q water at six concentration levels ranging from 0.01 to 2 µg L^−1^ and extracted using the same conditions applied to the samples. Each calibration level was injected in triplicate to ensure reproducibility. Limits of detection (LOD) and quantification (LOQ) were calculated based on signal–noise (S/N) ratios of 3 and 10, respectively.

## 3. Results and Discussion

Surveys were carried out during typical winter-time conditions characterised by energetic meteo-marine events and a limited affluence of beach users, which greatly limited the amount of litter abandoned by visitors [30]. Therefore, recollected litter consisted of items of different origin and composition recently stranded on the shore after spending an unknown period of time in the ocean. This was reflected by evident signs on beach litter items of prolonged exposure to saltwater and marine processes, such as discoloration, abrasion, and the presence of bryozoans, corals, or algae [30].

### 3.1. Litter Abundance and Characteristics

**Figure 3:**
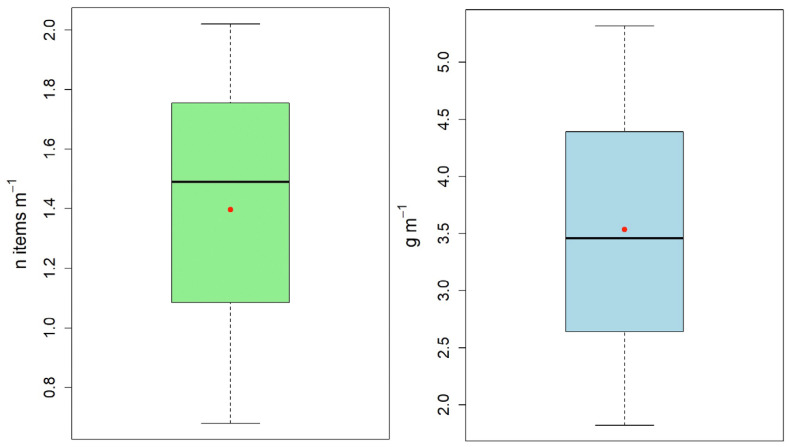
Boxplots of the total number of items (items m^−1^, on the left) and weight (g m^−1^, on the right) of beach litter recorded at Bajo de Guía, La Jara, and La Puntilla. The black horizontal line inside each box represents the median of the data, while the red dot indicates the mean.

A total of 1342 items were identified, with a cumulative weight of 3.4 kg over a total length of almost 900 m (Figure 3).

The beach with the highest litter abundance, i.e., number of items m^−1^, was Bajo de Guía (2.02 items m^−1^), followed by La Puntilla (1.49), and then La Jara (0.68). However, considering the total weight of litter density, it varied from 1.82 g of items m^−1^ at Bajo de Guía, to 3.46 at La Jara, and to 5.32 at La Puntilla.

The comparison of data on beach litter abundance obtained within different investigations is often very challenging due to the differences in methodologies used, the type of beach (e.g., remote vs. urban), and the period (e.g., summer vs. winter) of monitoring [41]. Considering other studies previously carried out at the investigated sites, the numbers of litter items recorded at Bajo de Guía and La Jara were very close to the values (2.11 and 0.96 items m^−1^) observed in October 2018 by Asensio-Montesinos et al. [35], and values observed at La Puntilla were very close to the values (1.36 items m^−1^ or 6.62 g m^−1^) recorded at that beach by Ciufegni et al. [30] in March 2023. Asensio-Montesinos et al. [35] classified the investigated beaches according to the Beach Grading [42] that considers the presence/absence and quantity of nine categories of litter items and allows the rating of each site from “A” (excellent quality, i.e., very clean site) to “D” grade (poor quality, i.e., very dirty site). Bajo de Guía was the most polluted site (“D” score) because of the presence of sewage-related items and general litter categories. La Jara was rated “B”, and La Puntilla obtained a “C” score. Therefore, the beaches investigated presented a medium to high level of pollution.

**Figure 4:**
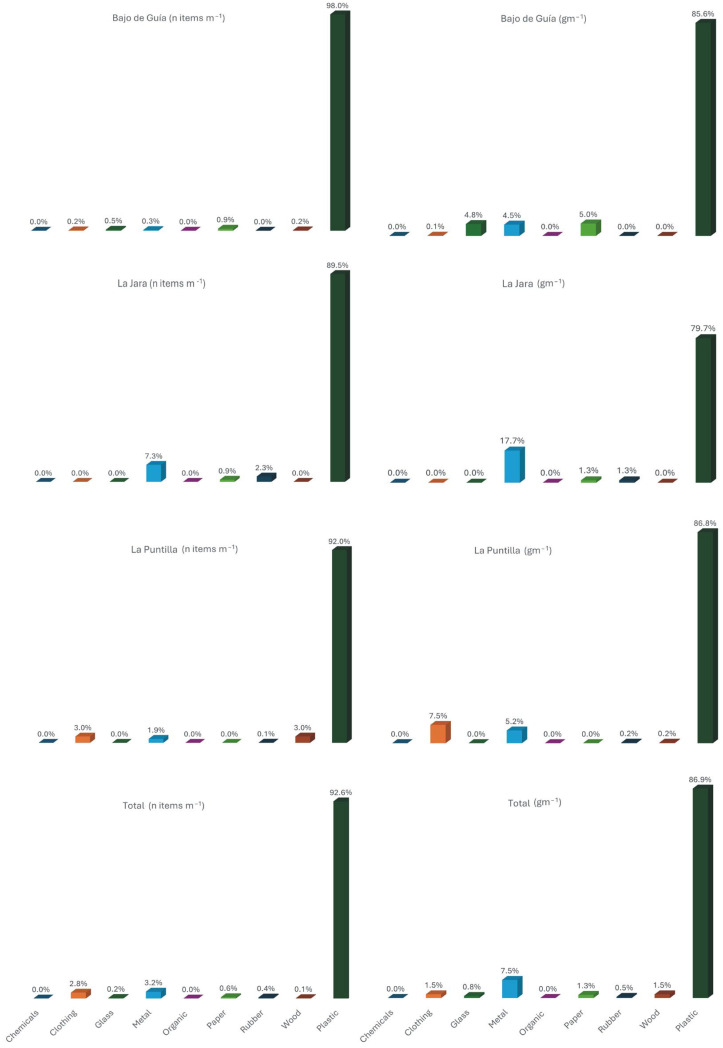
Total beach litter composition considering the number of items per meter (on the **left**) and the weight of items (on the **right**). The different percentages may not sum 100% because values were approximated to the first decimal.

Concerning litter composition (Figure 4), plastic was the most abundant material found across all beaches, accounting for 93% of the total number of items and for 87% of the total weight. This is in accordance with previous local studies, e.g., [30,35], that observed that plastic items represented 88% of total materials, and studies carried out by several authors on European beaches [43], in the southern coast of the Mediterranean [44], in the Adriatic Sea [45,46], in the Black Sea [47], in Chile [48], and in Africa [49]. The overwhelming abundance of plastic items is due to their lightweight nature, great use, durability, and low rates of recovery [50]. Further, their positive or neutral buoyancy favours their displacement at the water’s surface that is linked to wind-induced and Stokes drift currents [51].

Metal was the second most abundant material, representing 8% of the total weight and 3% of the total number of items. Wood was the third most prevalent material, contributing 2% of the total weight, while clothing represented 3% in terms of number of items.

Overall, observed concentrations reflected the results obtained at the local level by Asensio-Montesinos et al. and Ciufegni et al. [30,35], as well as other studies carried out at different localities [52,53,54,55,56,57].

**Figure 5:**
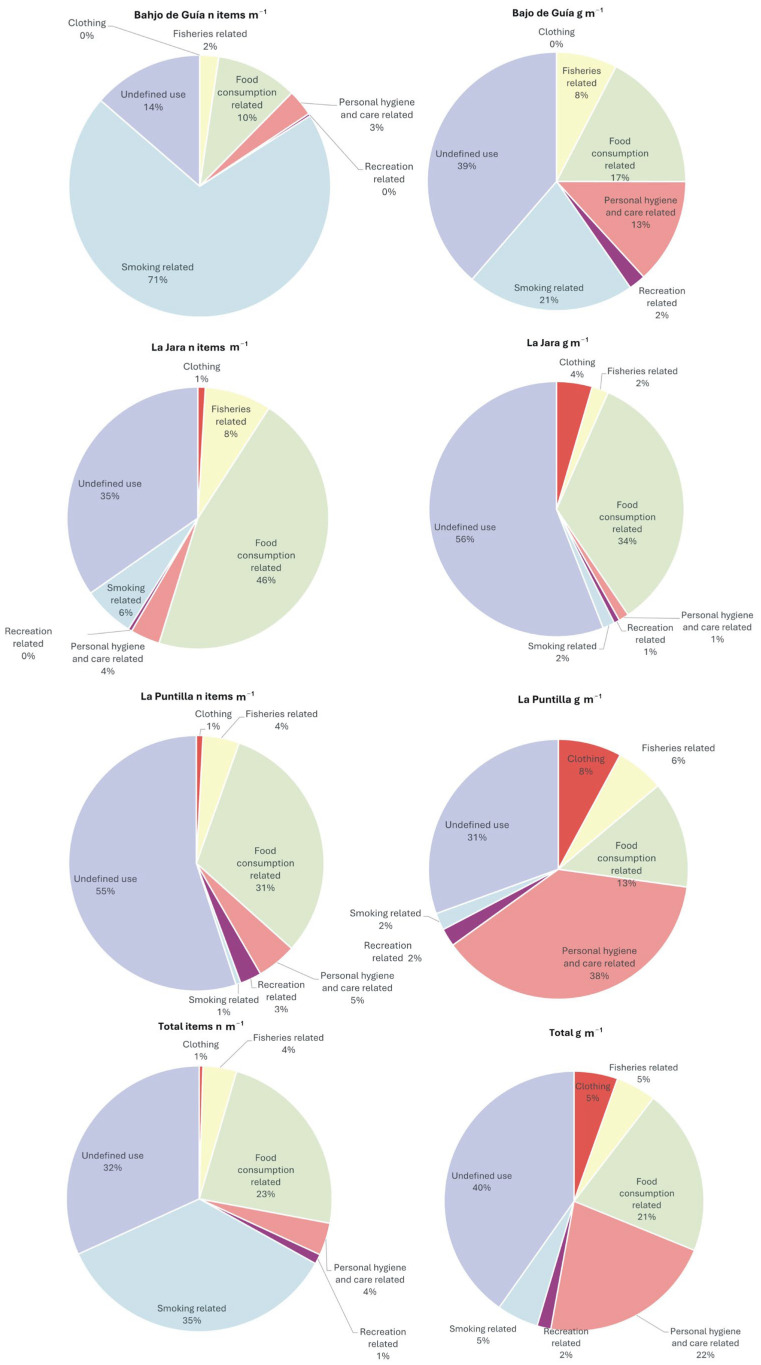
Beach litter classified for the three beaches according to use categories proposed by the Joint List of Litter Categories for Macrolitter Monitoring. Calculations are based on the number of items per meter (on the **left**) and on the weight of the items (on the **right**). The different percentages may not sum 100% because values were approximated to the first decimal.

Beach litter items were also categorised according to their usage (Figure 5). The largest portion consisted of smoking-related products, primarily due to the large number of cigarette butts found at Bajo de Guía. A significant portion of items, i.e., around one-third of the total, was classified under the “undefined use”, reflecting the high proportion of unidentifiable plastic pieces and other materials. The third major category was related to food consumption, which represented a notable share of the items, with crisps packets representing the most abundant element (Figure 5).

Differences were observed when the weight of items was considered (Figure 5). Undefined litter accounted for 40% of the total weight, followed by hygiene- and care-related products at 21% and food consumption-related items at 14%. This shift is largely attributable to the lightweight nature of cigarette butts that typically weigh significantly less than 1 g each. They constitute hazardous wastes that are not properly discarded by smokers at the beach and/or inland and often represent the most abundant litter items in the environment and a threat to various organisms because they leak numerous pollutants [58]. Indeed, the abundance of this type of personal waste has been very well-recognised all around the world [55,59,60,61,62] and is quite common on Spanish beaches, too [4,35,63,64]. According to Asensio-Montesinos et al. [35], the large amount of cigarette butts recorded at Bajo de Guía beach was mainly due to the inputs from the Guadalquivir River and the nearby public spaces, such as promenades, roads, and pavements, as observed at other places by Araújo and Costa [62]. Their accumulation in beach environments primarily occurs during summer months due to the great affluence of visitors and the low efficiency of clean-up operations that are not able to gather small items such as cigarette butts, plastic pieces, and bottle caps [60,65]. Within the “Personal hygiene and care related” category, numerous wet wipes and, secondarily, sanitary towels, especially at Bajo de Guía and La Puntilla, were observed. As observed by Williams et al. and Ciufegni et al. [30,32,66], they are common at the investigated beaches and represent potentially dangerous items since they are associated with wastewater discharges transported by the Guadalquivir and Guadalete rivers. Concerning the food consumption-related items, they are usually abandoned on the dry beach by visitors or transported by wind and runoff from nearby urbanised areas, and their abundance on the beaches investigated was previously recorded by Asensio-Montesinos et al. [35]. Ciufegni et al. [30] suggested that each item discarded on the beach not only contaminates the beach environment but also poses a high risk of entering into the ocean, and this is often the case for food wrappers, essentially represented on the beaches investigated by crisps packets. Such items move along the shore and are often stranded again on the beach, representing a relevant portion of the freshly deposited litter.

Last, the fishery-related litter category is composed of items such as plastic fishing lines, plastic floats for nets, and tangled fishing ropes, often lost or abandoned at the sea. They are relatively important along the studied beaches but can especially be found in rural/remote sites, as observed by Asensio-Montesinos et al. and Ciufegni et al. [30,35]. They have been also observed at many places around the world [67,68].

### 3.2. Identification of Composition from the Beach Litter from La Puntilla Beach by FTIR Analysis

All the selected fragments were analysed using the FTIR spectroscopy (see Supplementary Materials), except for the cigarette butts since they are composed of cellulose acetate [35].

The FTIR results showed that 33 out of the 34 fragments analysed were composed of synthetic polymers, and 1 was identified as cellulose. The FTIR spectra are shown in Figures S1 and S2 (Sample A and Sample B, respectively). Table 1 provides the specific details of each fragment analysed via FTIR, the spectral library used for identification, and the description of the best spectral match provided by the software.

The dust wipes and wet wipes were composed of polyethylene terephthalate (PET), and the sanitary towels were made of polyethylene (PE) (Table 1). The hard plastics category included a mixture of compounds (Table 1): five items consisted of PVC (42%); four items of PP (34%)–two of which were added with calcium carbonate; one item of PS; one item of PA (nylon); and one item of ABS (acrylonitrile–styrene–butadiene copolymer). The soft plastics category also included a mixture of compounds (Table 1): eight items of PET (42%), mostly polyester film and a thermoplastic elastomer; five items of PE (26%), including one of Neopolen foam, two of petrothene resins, one of HDPE, and one chlorosulfonated; two items of PVC, one of which was a clear extrusion compound; two items of PP (bag reinforced with polyol); one item of PA; and one item of cellulose.

In summary, PET and PVC were the most abundant compounds in terms of percentage, i.e., 29% and 20% respectively, but a relevant number of other polymers were also recorded. It is clear that polyolefin-based items (PP and PE) [69] were underrepresented among the objects selected for FTIR analysis. Obtained results are in accordance with previous local investigations [35]. In contrast to the findings of the present study, plastic items analysed in Gazi Bay (southern coast of Kenya) were found to consist of 42.62% PP, 35.95% PE, and only 14.83% PET; PS and PA were identified in amounts below 2%, while PVC, PE, and other polymers were present at concentrations below 1% [70]. As highlighted in a recent review [71], the macro litter items are generally identified based on qualitative characteristics, such as size, type, usage categories, etc. When FTIR is performed, only a fraction of the collected items is analysed with this technique [71,72,73], and meaningful comparison of the available macro beach litter studies is substantially limited. Nevertheless, it is widely recognised most marine debris consists of plastic items made from various polymers, including polypropylene (PP), polyethylene (PE), polystyrene (PS), polyvinyl chloride (PVC), polyethylene terephthalate (PET), and polyamide (PA) [74,75,76]. The density of these compounds ranges from 0.9 to 1.5 g/cm^3^, a characteristic that influences their buoyancy behaviour in the marine environment [77,78]. The majority of synthetic polymers, such as PP and PS, are buoyant in the ocean, whereas others sink, such as high-density polyethylene (HDPE) and PET [79]. The density of coastal seawater (1.02 g/cm^3^) is higher than the density of PE and PP (0.9—1.0 g/cm^3^) [79]; therefore, items composed of such materials show positive buoyancy and are susceptible to being easily transported far away by currents and waves and eventually strand on distant beaches, whereas items made of denser materials such as PVC and PET tend to settle in deeper waters, close to their point of release.

These observations align with the findings of this paper. Indeed, items composed of PET (such as dust wipes and wet wipes, related to the “personal hygiene” category, and food wrappers, related to the “food consumption” category, which are very relevant in the study area; see Figure 5) and PVC (such as a freshener package, a medicine blister pack, and a corrugated tube) are related to land-based activities. Such items could have been easily deposited on the strandline via the nearby Guadalete River’s mouth as previously stated by Williams et al. [80] and observed elsewhere by other authors [80,81]. A similar fate can be attributed to sanitary towels. Although made of PE, their structure tends to absorb water, sediment, and other materials, substantially altering their buoyancy and thus explaining the high concentration of this type of item on the strandline. As for the other PE items (such as fragments of bags and packages) and PP items (such as plastic pieces and films), it is not possible to identify a single source of release, as all of these objects can enter coastal environments through multiple pathways [53,82].

A recent study examining the stomach contents of stranded alcids on the coasts of Andalusia (Spain) identified the following six polymer types using μ-FTIR spectral analysis: polyethylene (PE), polyamide (PA), polypropylene (PP), polyethylene terephthalate (PET), styrene allyl alcohol (SAA), and regenerated cellulose [83].

It is known that plastic polymers contain more than 400 added compounds [84,85], dependent on their use, to adjust their characteristics or to enhance stabilisation against environmental exposure, raising considerable environmental concerns [86,87]. Certain polymers may contain substantial quantities of different additives (e.g., antioxidants, plasticisers, and flame retardants) or fillers to reduce overall costs and modify mechanical and functional properties [88,89,90,91]. Even though polymers are typically considered unreactive materials, chemical additives like phthalates and bisphenols can be released, permeating the marine environment [86,92,93,94]. The most evident impacts on marine environments include the leaching of chemicals and plastic additives (i.e., pigments, plasticisers, fillers, stabilisers, antioxidants, and flame retardants) [95]. Moreover, these impacts are intensified by the risks associated with the adsorption of additional compounds, potentially enhancing environmental concentrations of chemical contaminants and their propensity for bio-accumulation and bio-magnification within the trophic web, this process resulting in further hazards for marine fauna and human health [96,97,98,99,100,101]. Consequently, subsequent to identifying the primary polymers within the different categories, this study investigated ex situ leaching experiments in order to identify contaminants released from the selected plastic items that were part of their formulation or were previously adsorbed during their time spent in the oceans.

### 3.3. Identification and Quantification of Organic Compounds

**Figure 6:**
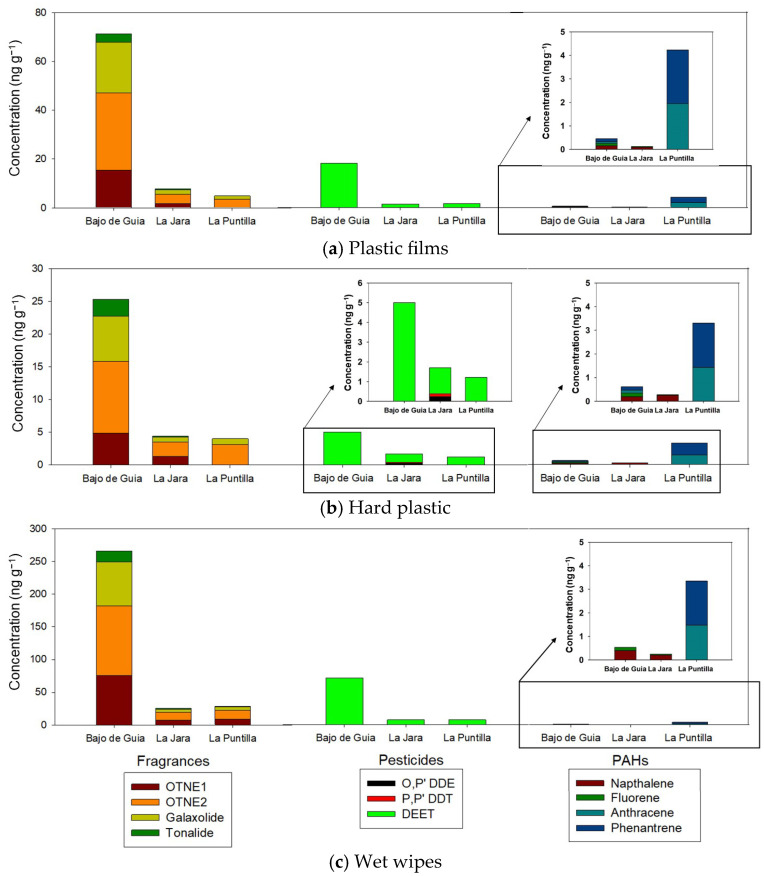
Concentration of organic pollutants measured on leaching experiments of (**a**) plastic films, (**b**) hard plastics, and (**c**) Wet wipes collected on the Bajo de Guía, La Jara and La Puntilla beaches.

The data on adsorbed compounds presented in this investigation are based on a single survey of plastic stranded beach litter items; therefore, in order to have more representative and general results, further investigations have to be carried out and based on several plastic beach litter surveys. Figure 6 shows the organic compounds leached from the plastic films (Figure 6a), hard plastics (Figure 6b), and wet wipes (Figure 6c) collected from the three beaches under study. As the concentration and pollution levels follow the same trends, these data are presented together. The different pollutants are categorised as fragrances, pesticides, insect repellents, or PAHs.

The most abundant compounds were fragrances, reaching concentrations of 25 ng g^−1^ to 106.60 ng g^−1^ for OTNE2, 1.28 ng g^−1^ to 75.50 ng g^−1^ for OTNE1, 0.7 ng g^−1^ to 67.26 ng g^−1^ for galaxolide, and 0.18 ng g^−1^ to 16.96 ng g^−1^ for tonalide. OTNE1 and OTNE2 are the most widely used fragrances; some authors have calculated discharges of up to 0.61–1.9 g y^−1^ per person in Germany [102]. Furthermore, both galaxolide and tonalide have been identified as toxic marine compounds that induce oxidative stress and genotoxicity [103]. The hydrophobic characteristic of these compounds indicates tendency to remain adsorbed on plastic films or in sediments [104].

Concerning pesticides and insect repellents, Bajo de Guia was the most polluted place, with DEET being the most frequently detected compound, with concentrations of up to 71.68 ng g^−1^. DEET is a widely used insect repellent against mosquitoes, and it is often detected in many aquatic systems, including wastewater treatment plant effluent and treated drinking water [105,106]. Therefore, it is a highly recalcitrant compound as it is both persistent and mobile through different environmental systems.

Concerning O,P’-DDE and O,P’-DDT, concentrations of 0.25 and 0.15 ng/g were found at La Jara beach. Due to the hydrophobic properties of these compounds, marine litter can act as an important sink for these substances. Some authors have detected them in plastics on the seabed, and they may be desorbed into marine sediments [24].

**Figure 7:**
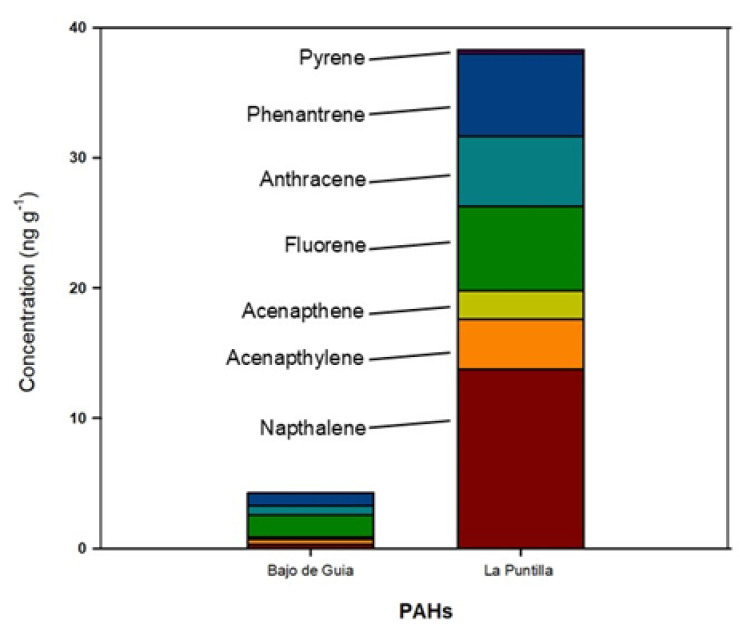
Concentration of PAH identified during the leaching experiments on the smoking-related items—cigarettes butts—collected on the Bajo de Guia and La Puntilla beaches.

Different PAH species were also identified; anthracene and phenanthrene were the most abundant at La Puntilla beach, while naphthalene was the most abundant at the Bajo de Guia and La Jara beaches. Moreover, a lower concentration was encountered in these leachates (Figure 6) than concentrations found in smoking-related items (Figure 7). This suggests that these compounds have different origins and that PAHs may be adsorbed by different types of plastic debris [107]. Many studies focus on determining diagnostic ratios to model PAHs sources [107,108]. However, the different ageing of the items impedes the correct prediction of the origin of this pollution due to the different reactivity of the PAHs with environmental oxidative stress (UV and reactive oxygen species) [107]. Pollutants’ concentrations associated with wet wipes at the investigated beaches are presented in Table 2 and expressed as ng/m, a value obtained after considering the total weight of wet wipes collected at each beach and the total length of each surveyed beach.

Figure 7 illustrates the compounds identified in the smoking-related items from the Bajo de Guia and La Puntilla beaches, and their concentrations (ng/m) are presented in Table 3. Data for La Jara beach are not presented since not enough cigarette butts were found for analysis (see Figure 5). It can be observed that polycyclic aromatic hydrocarbons (PAHs) are the predominant compounds detected in this litter category. Furthermore, their concentrations were significantly higher at La Puntilla beach (38.33 ng g^−1^) compared to Bajo de Guía (4.26 ng g^−1^).

These compounds are released during the combustion organic matter [108,109] and are considered highly toxic due to their mutagenic, teratogenic, and carcinogenic properties [108]. Generally, PAHs are volatile compounds, and their reactivity to environmental factors—primarily UV radiation—varies depending on the specific compound [107,108]. Consequently, each compound exhibits a different environmental fate, governed by its physicochemical properties. Based on the data obtained, it can be inferred that PAH concentrations are indicative of the degree of ageing of cigarette butts, with higher concentrations associated with more recent disposal.

## 4. Conclusions

Plastic debris constitutes relevant vectors of persistent and emerging organic pollutants in the coastal environments. Adsorbed compounds range according to the category of analysed beach litter. The main findings of this preliminary study, which is based on a single survey of plastic stranded beach litter items, can be summarised as follow:

- Smoking-related materials contain high amounts of PAHs, especially at La Puntilla beach.
- Personal care products (PCPs), such as the wet wipes collected and used for desorption experiments, are a significant source of fragrances in the coastal environment. Concentrations of these compounds were one order of magnitude higher than in the rest of the analysed plastic litter categories. Additional co-leaching or sorption experiments must be done to prove the possible migration of fragrances from wipes to the rest of the materials.
- Plastic debris found at Bajo de Guia beach contained the highest concentrations of fragrances, pesticides, and insect repellent.
- The sources of contamination on the studied beaches are probably different.

This paper has identified the contaminants associated with the plastic debris commonly found on beaches in southern Spain and represents one of the first efforts to examine organic pollutants adsorbed onto different types of waste. It provides a valuable foundation for future research that includes the assessment of the degradation of recorded compounds under different environmental conditions and their possible migration to other environmental compartments, such as water, sediment, or biota.

## Figures and Tables

**Table 1:** Specifications of individual macro-litter fragments analysed by FTIR, detailing item name, sample and fragment identifiers, assigned functional groups based on FTIR spectra, the utilised spectral library, and the description of the closest spectral match.

Item(s)	Sample	Fragment	FTIR Group	Search Library	Search Best Hit Description
Dust wipes	A	1	PET	W	W01904.SP W01904 MELINEX 377/200 POLYESTER FILM TRANSLUCENT MATTE FILM
Wet wipes	A	2	PET	W	W01904.SP W01904 MELINEX 377/200 POLYESTER FILM TRANSLUCENT MATTE FILM
Sanitary towels	A	3	PE	W	W01468.SP W01468 PETROTHENE LR 923 POLYETHYLENE RESIN
Credit card	A	4	PVC	W	W01532.SP W01532 DURAL 600 POLYVINYL CHLORIDE
Cable tie	A	5	PA	POLIMERI	PA 6/6—NYLON 6/6 (POLY HEXAMETHYLENE ADIPAMIDE)
Transparent wardrobe freshener package	A	6	PVC	polyatr	POLY(VINYL CHLORIDE), CARBOXYLATED 1.8% CARBOXYLATED
Clear blue glass	A	7	PP	W	W01161.SP W01161 REXENE PP11S5
Medicine blister pack	A	8	PVC	polyatr	POLY(VINYL CHLORIDE) INHERENT VISCOSITY 1.26
Fork	A	9	PS	POLIMERI	POLYSTYRENE—UATR SPECTRUM
Green clothes peg	A	10	PP	W	W01448.SP W01448 FORTILENE 3251 POLYPROPYLENE
Blue hard plastic tube	A	11	ABS	POLIMERI	ABS—ACRYLONITRILE-STYRENE-BUTADIENE COPOLYMER—UNIVERSAL ATR
White piece of plastic	A	12	PP	POLIMERI	POLYPROPYLENE + CALCIUM CARBONATE
Corrugated tube	A	13	PVC	W	W01534.SP W01534 DURAL 602 NATURAL POLYVINYL CHLORIDE
Black piece of plastic	A	14	PP	POLIMERI	POLYPROPYLENE + CALCIUM CARBONATE
Creamy white soft tube	A	15	PVC	W	W01543.SP W01543 PVC 2214-85 CLEAR POLYVINYL CHLORIDE EXTRUSION COMPOUN
Inner layer of tetrapack package	B	1	PE	W	W02032.SP W02032 NEOPOLEN 1720 MOLDABLE POLY-ETHYLENE FOAM
Starlight package for fishing	B	2	PE	W	W01468.SP W01468 PETROTHENE LR 923 POLYETHYLENE RESIN
Green and black sweet wrapper	B	3	PET	W	W01904.SP W01904 MELINEX 377/200 POLYESTER FILM TRANSLUCENT MATTE FILM
Condom package	B	4	PA	polyatr	POLYAMIDE RESIN MELTING PT 95DEG C
Small gray bag	B	5	PET	W	W01904.SP W01904 MELINEX 377/200 POLYESTER FILM TRANSLUCENT MATTE FILM
Yellow piece of plastic	B	7	PE	polyatr	POLYETHYLENE HIGH DENSITY
Blue adhesive label	B	8	PET	W	W01904.SP W01904 MELINEX 377/200 POLYESTER FILM TRANSLUCENT MATTE FILM
Transparent film (n. 1, in Figure 2)	B	9	PET	polyatr	POLY(ETHYLENE THEEPHTHALATE)
Transparent and purple sweet wrapper	B	10	PET	W	W01904.SP W01904 MELINEX 377/200 POLYESTER FILM TRANSLUCENT MATTE FILM
White and blue sweet wrapper	B	11	PET	W	W01904.SP W01904 MELINEX 377/200 POLYESTER FILM TRANSLUCENT MATTE FILM
Transparent film (n. 2, in Figure 2)	B	12	PP	W	W01972.SP W01972 NOVA-PAC POLYPROPYLENE SEED BAG REINFORCED WITH POLYOL
Fragment of white bag	B	13	PE	polyatr	POLYETHYLENE, CHLOROSULFONATED
Transparent straw wrapper	B	14	PP	W	W01972.SP W01972 NOVA-PAC POLYPROPYLENE SEED BAG REINFORCED WITH POLYOL
Brown piece of plastic	B	15	PVC	W	W01543.SP W01543 PVC 2214-85 CLEAR POLYVINYL CHLORIDE EXTRUSION COMPOUN
Transparent film (n. 3, in Figure 2)	B	16	PET	W	W01904.SP W01904 MELINEX 377/200 POLYESTER FILM TRANSLUCENT MATTE FILM
Fragment of green bag	B	17	Noplastic	POLIMERI	CELLULOSE
Outer layer of tetrapak	B	18	PE	W	W01468.SP W01468 PETROTHENE LR 923 POLYETHYLENE RESIN
Fragment of blue bag	B	19	PVC	W	W01215.SP W01215 ALPHA PVC 2212-100 CLEAR POLY- VINYL CHLORIDE
Black electrical tape	B	20	PET	POLIMERI	THERMOPLASTIC ELASTOMER POLYESTER BASE—HYTREL

**Table 2:** Pollutants’ concentrations associated with wet wipes at the investigated beaches.

Site	Concentrations (ng m^−1^)		
	PAHs	Pesticides	Fragrances
La Jara	0.01	0.31	1.03
Bajo de Guía	0.07	7.66	28.47
La Puntilla	11.61	26.84	100.50

**Table 3:** Pollutants’ concentrations associated with cigarette butts at the investigated beaches.

Site	Concentrations (ng m^−1^)		
	PAHs	Pesticides	Fragrances
Bajo de Guía	1.06	4.52	26.66
La Puntilla	2.75	19.03	0.33

## Data Availability

The data presented in this study are available on request from the corresponding author.

## Supplementary Materials

The following supporting information can be downloaded at: https://www.mdpi.com/article/10.3390/toxics13080673/s1, Figure S1: FTIR spectra of compounds identified among the analysed fragments on sample A; Figure S2: FTIR spectra of compounds identified among the analysed fragments on sample B.

## Abbreviations

The following abbreviations are used in this manuscript:

DDE	Dichlorodiphenyldichloroethylene
DDT	Dichlorodiphenyltrichloroethane
DEET	N,N-Diethyl-Meta-Toluamide
DOC	Dissolved organic carbon
EHMC	2-Ethylhexyl-4-Methoxycinnamate
FTIR	Fourier Transform Infrared Spectroscopy
GC—MS	Gas Chromatography—Mass Spectrometry
LOD	Limits of detection
LOQ	Limits of quantification
LS	Liquid–solid
MBC	Methylbenzylidene Camphor
MRM	Multiple reaction monitoring
MSFD	Marine Strategy Framework Directive
MTCS	Methyl-Triclosan
OTNE	Tetramethyl Acetyloctahydronaphthalenes
PAHs	Polycyclic Aromatic Hydrocarbons
PCBs	Polychlorinated Biphenyls
PDMS	Polydimethylsiloxane
PE	Polyethylene
PET	Polyethylene terephthalate
POPs	Persistent organic pollutants
PP	Polypropylene
PS	Polystyrene
PVC	Polyvinyl Chloride
S/N	Signal–noise
SBSE	Stir bar sorptive extraction
SW	Southwest
TCS	Triclosan
UV	Ultraviolet
