# Supplementary material for: Assessment of Organic Pollutants Desorbed from Plastic Litter Items Stranded on Cadiz Beaches (SW Spain)
Source: Toxics. 2025 Aug 9;13(8):673. doi: 10.3390/toxics13080673 (PMC12389833; doi:10.3390/toxics13080673)
Supplement: Supplementary file 1 [file toxics-13-00673-s001.zip › toxics-3780210-supplementary.pdf]

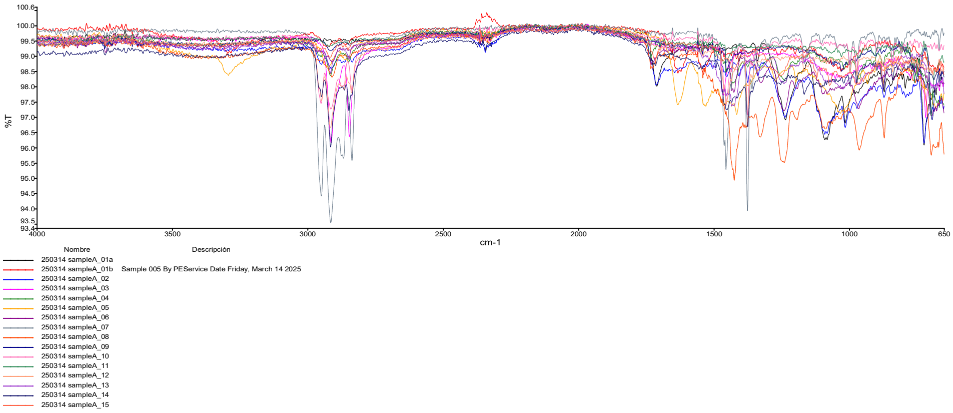

Figure S1. FTIR spectra of compounds identified among the analysed fragments on sample A.

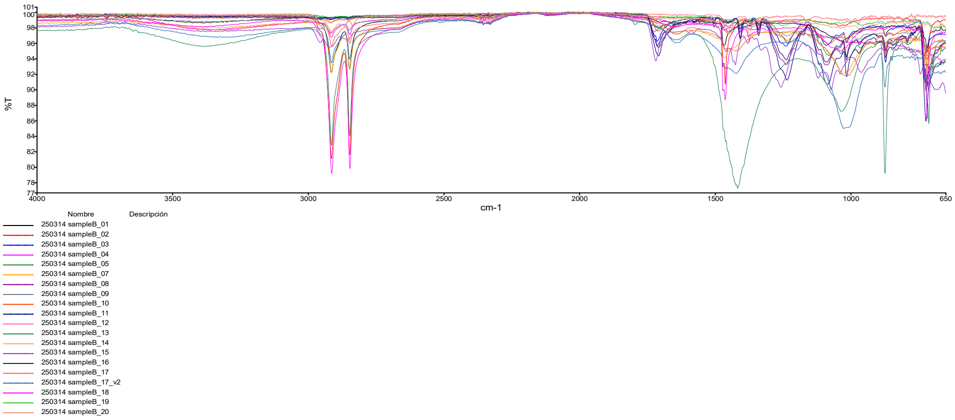

Figure S2. FTIR spectra of compounds identified among the analysed fragments on sample B
